# Herptile gut microbiomes: a natural system to study multi-kingdom interactions between filamentous fungi and bacteria

**DOI:** 10.1128/msphere.00475-23

**Published:** 2024-02-13

**Authors:** Lluvia Vargas-Gastélum, Alexander S. Romer, Marjan Ghotbi, Jason W. Dallas, N. Reed Alexander, Kylie C. Moe, Kerry L. McPhail, George F. Neuhaus, Leila Shadmani, Joseph W. Spatafora, Jason E. Stajich, Javier F. Tabima, Donald M. Walker

**Affiliations:** 1 Department of Botany and Plant Pathology, Oregon State University, Corvallis, Oregon, USA; 2 Department of Biology, Middle Tennessee State University, Murfreesboro, Tennessee, USA; 3 Research Division 3, Marine Ecology, GEOMAR Helmholtz Centre for Ocean Research Kiel, Kiel, Germany; 4 Department of Pharmaceutical Sciences, College of Pharmacy, Oregon State University, Corvallis, Oregon, USA; 5 Department of Microbiology and Plant Pathology, University of California Riverside, Riverside, California, USA; 6 Institute for Integrative Genome Biology, University of California, Riverside, California, USA; 7 Department of Biology, Clark University, Worcester, Massachusetts, USA; University of Wisconsin-Madison, Madison, Wisconsin, USA

**Keywords:** amphibian, reptile, anaerobic gut fungi, mycobiome, cyclic peptide, specialized metabolite, non-ribosomal peptide synthetases

## Abstract

Reptiles and amphibians (herptiles) are some of the most endangered and threatened species on the planet and numerous conservation strategies are being implemented with the goal of ensuring species recovery. Little is known, however, about the gut microbiome of wild herptiles and how it relates to the health of these populations. Here, we report results from the gut microbiome characterization of both a broad survey of herptiles, and the correlation between the fungus *Basidiobolus*, and the bacterial community supported by a deeper, more intensive sampling of *Plethodon glutinosus*, known as slimy salamanders. We demonstrate that bacterial communities sampled from frogs, lizards, and salamanders are structured by the host taxonomy and that *Basidiobolus* is a common and natural component of these wild gut microbiomes. Intensive sampling of multiple hosts across the ecoregions of Tennessee revealed that geography and host:geography interactions are strong predictors of distinct *Basidiobolus* operational taxonomic units present within a given host. Co-occurrence analyses of *Basidiobolus* and bacterial community diversity support a correlation and interaction between *Basidiobolus* and bacteria, suggesting that *Basidiobolus* may play a role in structuring the bacterial community. We further the hypothesis that this interaction is advanced by unique specialized metabolism originating from horizontal gene transfer from bacteria to *Basidiobolus* and demonstrate that *Basidiobolus* is capable of producing a diversity of specialized metabolites including small cyclic peptides.

**IMPORTANCE:**

This work significantly advances our understanding of biodiversity and microbial interactions in herptile microbiomes, the role that fungi play as a structural and functional members of herptile gut microbiomes, and the chemical functions that structure microbiome phenotypes. We also provide an important observational system of how the gut microbiome represents a unique environment that selects for novel metabolic functions through horizontal gene transfer between fungi and bacteria. Such studies are needed to better understand the complexity of gut microbiomes in nature and will inform conservation strategies for threatened species of herpetofauna.

## INTRODUCTION

Approximately 21% of evaluated reptiles and 41% of amphibians are threatened with extinction (1), thus adversely impacting species diversity and ecosystem services (2, 3). Given the large threat to biodiversity, active conservation strategies are currently being utilized, including captive breeding programs, establishment of assurance populations and wildlife corridors, and translocation of individuals to enhance population genetics (4); however, the gut microbiome has yet to be broadly incorporated into wildlife conservation, due to a lack of knowledge, the complexity of manipulating microbial communities, and the effect of this intervention in host health (5). Pathogen-induced dysbiosis (6), habitat degradation (7, 8), and climate change (9, 10) are all linked to alterations in the microbiome with potential for adverse consequences to the host organism. In-depth knowledge of fungal-bacterial interactions in the herptile gut microbiome is therefore necessary to establish a baseline understanding of these understudied systems.

While herptile-fungal interactions on amphibian skin have been the subject of recent focus due to chytridiomycosis (11–13), the gut microbiome of herptiles remains understudied compared to other groups of animals. Only a few studies have simultaneously focused on the herptile gut microbiome in more than one domain of life (i.e., bacteria; e.g., references 14–16). Limiting the focus to the bacterial community results in overlooked fungal-bacterial interactions which may have diverse outcomes for the composition and function of the gut microbiome and, ultimately, influence host health (17).

Although as many as 50 genera of fungi have been documented in the human gut, fungi—mainly yeasts, make up a relatively small proportion of the human gut microbiome (18, 19). Even less is known about fungi inhabiting the digestive systems of wildlife species. For example, the obligate anaerobic gut fungi (AGF), which are ubiquitously distributed among herbivorous ruminant animals and are essential to the digestion of lignocellulosic plant fiber, were only recently discovered in 1975 (20–22). Many herptiles are not herbivorous and instead feed on invertebrates like insects. Feeding strategy is predictive of the gut microbiome since herbivorous herptiles host different assemblages of gut microbiota compared to insectivores, including from the fungal genus *Basidiobolus* (15).

**Fig 1:**
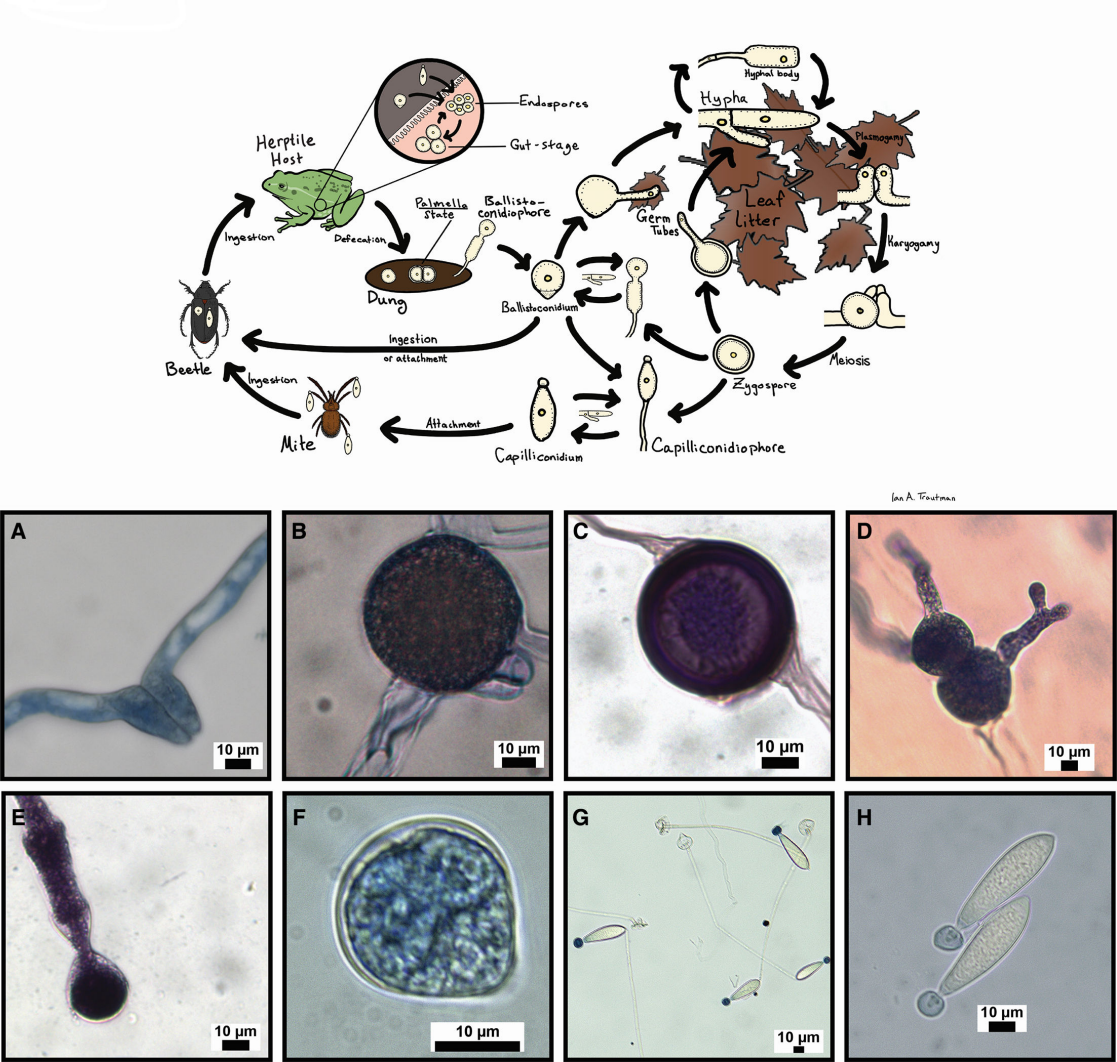
*Basidiobolus* life cycle and spore morphologies. Schematic of *Basidiobolus* life cycle showing major spore and vegetative stages and representation of spore morphologies: (A) compatible hyphae prior to zygospore formation, (**B**) young zygospore, (**C**) mature zygospore, (**D**) zygospores germinanting, (**E**) ballistoconidiophore, (**F**) ballistoconidium, (**G**) capilloconidiophores, and (**H**) capilloconidia.

*Basidiobolus* is a filamentous, gut-inhabiting fungus isolated from the feces of a wide diversity of herptile hosts (16, 23, 24). Resting spores of *Basidiobolus* are dispersed in fecal pellets, and upon defecation, germinate to produce hyphae and a diversity of spore types (Fig. 1). Hyphae grow and develop into a vegetative thallus (mycelium), and in many species produce sexually reproductive gametangia which fuse to form zygospores. Hyphae also give rise to conidiophores that produce apical, forcibly discharged asexual primary spores, blastoconidia, that germinate and give rise to a mycelium, or other spore types including capilloconidia. Capilloconidia possess adhesive tips that adhere to the exoskeletons of passing insects. These insects are eventually consumed by insectivorous hosts, reinoculating the host animal and completing the life cycle (Fig. 1). The broad metazoan host diversity (anurans, bats, fishes, lizards, salamanders, snakes, turtles, and wallabies (23, 25–29), a unique lifecycle, and the presumption that *Basidiobolus* has acquired appreciable amounts of genes through horizontal transfer (HGT) from co-occurring gut bacteria, make this an interesting system to study bacterial-fungal interactions in herptile gut microbiomes.

AGF represent the most well-documented case of HGT in fungi (30, 31). AGF genomes have a documented HGT rate of 2.0–3.5%, allowing AGF to expand substrate utilization range, diversify pathways for electron disposal, acquire novel secondary metabolism, and facilitate adaptation to the anaerobic environment (30). Phylogenomic analyses of three *Basidiobolus* genomes reveal a similar magnitude of HGT to AGF (32). *Basidiobolus* is a nonflagellated, filamentous fungus that last shared a common ancestor with AGF ~700 million years ago (MYA) (33). It is phylogenetically related to a diversity of “zygomycete” fungi (34) that associate with aquatic stages of arthropods, nematodes, rotifers, amoeba, and other fungi in relationships ranging from parasitic to commensal. *Basidiobolus* is the only known group of fungi that are specialized to the herptile gut and represents an independent origin of gut fungi as compared to the AGF.

This work is focused on understanding the diversity of bacteria and fungi in the gut of herptiles, interactions between *Basidiobolus* and other gut bacteria and fungi, genome signatures of HGT in *Basidiobolus*, and the suite of metabolites produced by species of *Basidiobolus*. We characterize the gut microbiome of 33 species of frogs, lizards, and salamanders and show differences across both host and geographic regions. We expanded the phylogenetic diversity of living *Basidiobolus* cultures and documented signatures of host and geographic preference and co-colonization of more than one putative species of *Basidiobolus* in the gut of herptile individuals. Network and indicator species analyses suggest correlations between *Basidiobolus* and other gut fungi and bacteria. Phylogenomic analysis of *Basidiobolus* indicated 2–5% of genes predicted to be of bacterial origins with enrichment of genes coding for specialized metabolism. Non-targeted LC-MS/MS and network analysis revealed peptidic metabolite signatures produced by cultures of *Basidiobolus* and in the gut of herptiles. We discuss herptile gut microbiomes in the context of fungal adaptations to the animal gut microbiome environment and microbial interactions between filamentous fungi and bacteria.

## RESULTS AND DISCUSSION

### Herptile microbiomes are characterized by unique bacterial and filamentous fungal communities

**Fig 2:**
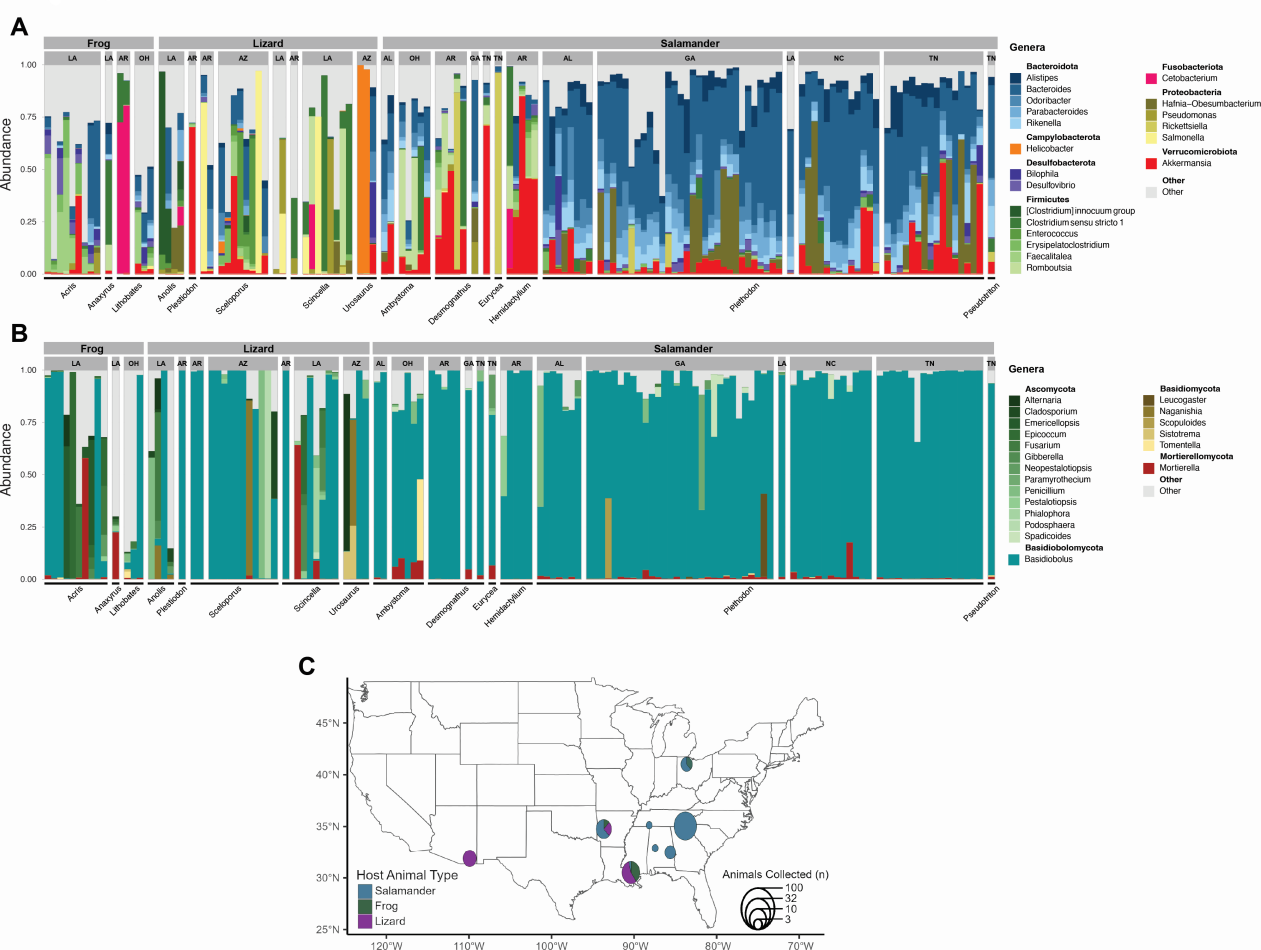
Taxonomic composition of the 20 most abundant genera of the herptile gut microbiome. Fungi (**A**) and bacteria (**B**) found in the gut of frogs, lizards, and salamanders from the different sampled geographic locations: Alabama (AL), Arkansas (AR), Arizona (AZ), Georgia (GA), Louisiana (LA), North Carolina (NC), Ohio (OH), and Tennessee (TN). Herptile genera are listed on the *x*-axis of panels A and B. (**C**) Map of collection locations, sample sizes, and host affinity of each collection; sites in close proximity (i.e., eastern TN, western NC, and GA) were pooled.

The rarefied data set consisted of 133 herptiles (Table S1) from eight different states in the United States (Arizona, Ohio, North Carolina, Tennessee, Georgia, Alabama, Arkansas, and Louisiana; Fig. 2C). A total of nine samples were removed from the 16S rRNA and ITS rDNA data sets due to quality control filtering. After quality control, DADA2 processing, decontamination and rarefaction, a total of 9,196,775 fungal ITS1 rDNA sequences and 6,110,012 bacterial 16S rRNA sequences were retained. These sequences resulted in a total of 5,562 fungal ASVs and 9,343 bacterial ASVs. Nine fungal and 35 bacterial phyla were identified. The most abundant phyla were Ascomycota, Zoopagomycota, and Basidiomycota for fungi, and Bacteroidota, Firmicutes, and Proteobacteria for bacteria (Fig. 2).

Differences were observed when comparing relative abundance data among hosts for both bacteria and fungi (Fig. 2). For bacteria, the most obvious difference was the differential representation of Bacteriodota and Firmicutes as a function of host grouping of frogs, lizards, and salamanders. Firmicutes represented 64.7% of the abundance in frogs; Firmicutes represented 39.7% and Proteobacteria 39% of the abundance in lizards; and Bacteroidota accounted for 40.1% of the abundance in salamanders. Relative abundances of salamander gut bacteria were mostly similar across geographic locations, except for Arkansas and some Ohio sites, where they were dominated by Firmicutes and *Akkermansia* (Fig. 2A).

For fungi, *Basidiobolus* represented the most abundant genus in salamanders and lizards with values of 84.4% and 52.8%, respectively. In frogs, genera of the Ascomycota were collectively the most abundant group (44.2%), followed by *Basidiobolu*s (29%). *Basidiobolus* dominated the fungal composition in the majority of salamander samples, ranging from >60% to 99% of the abundance, and little variation in fungal communities across different geographic locations. Frog and lizard samples were more variable across geographic localities, as the majority of samples were dominated by *Basidiobolus*, or other genera of Ascomycota (*Alternaria*, *Emericellopsis*, *Epicoccum*, and *Penicillium*) and Basidiomycota (*Naganishia* sp.; Fig. 2B). Several other species of fungi have been previously documented in the gut of herptiles including *Aspergillus fumigatus*, *Geotrichum candidum*, *Trichosporon* sp., and *Candida parapsilosis* (23). In a metabarcoding and high-throughput ITS rDNA sequencing study (15), *Basidiobolus ranarum* and *Basidiobolus magnus* dominated the core fecal mycobiome of *Sceloporus grammicus* lizards. They also documented *Aureobasidium microstictum*, *Hyphopichia burtonii*, *Penicillium thomii*, *Talaromyces duclauxii*, and *Tetraspisispora fleetii* as members of the lizard mycobiome. All of the previous genera, with the exception of *Geotrichum*, *Hyphopichia*, and *Tetraspisispora* were also found in this study.

**Fig 3:**
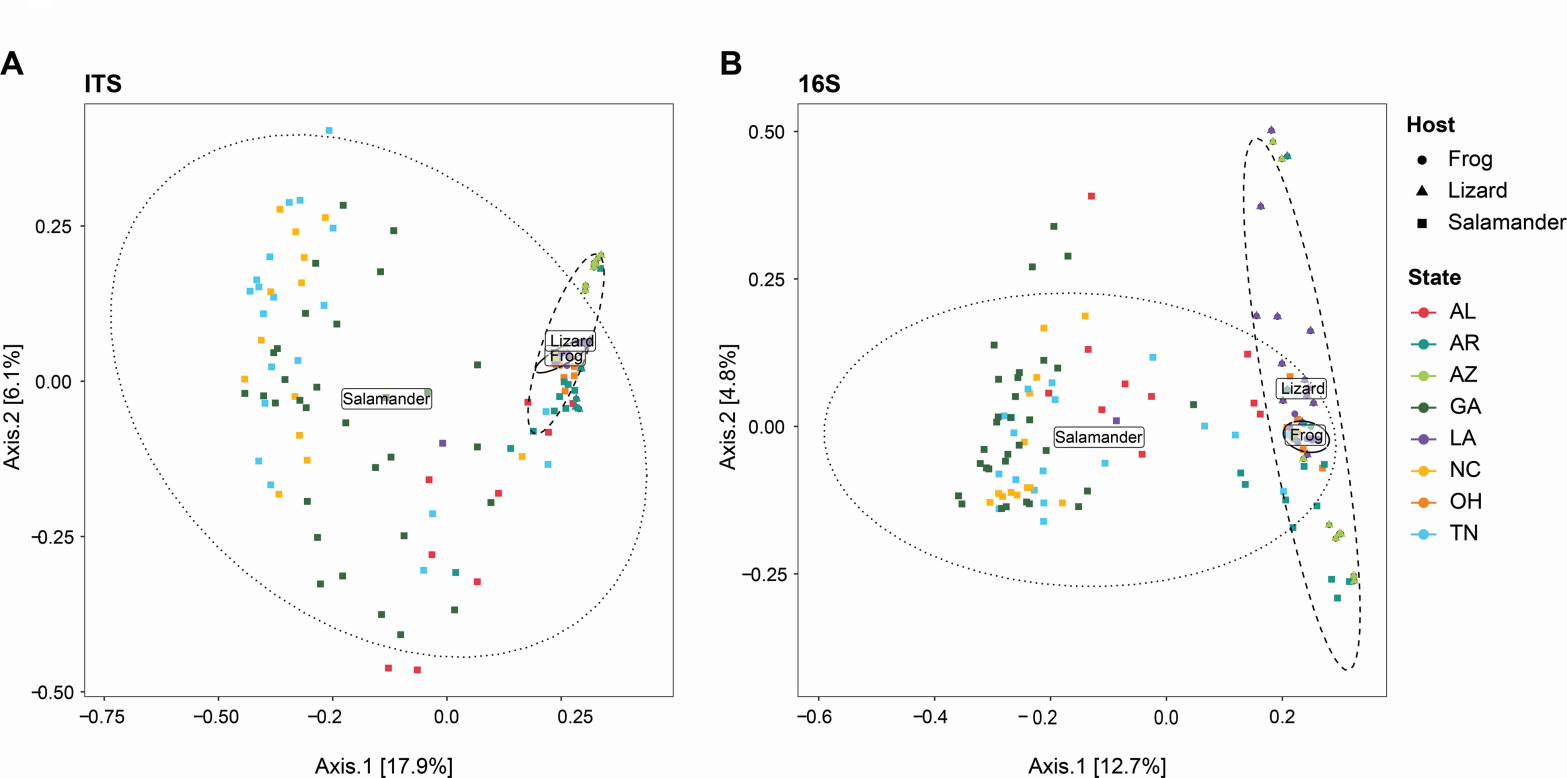
PCoA of fungal and bacterial communities based on Bray-Curtis dissimilarity. Host and geographic diversity of gut fungi (**A**) and bacterial (**B**) assemblages. Locations: Alabama (AL), Arkansas (AR), Arizona (AZ), Georgia (GA), Louisiana (LA), North Carolina (NC), Ohio (OH), and Tennessee (TN).

The PERMANOVA assumption of homogeneity of variance was violated when comparing the multivariate dispersion of host groups for both bacteria (betadisper; *F*_2, 132_ = 8.205, *P* = 0.002) and fungi (betadisper; *F*_2, 132_ = 10.026, *P* = 0.001). However, PERMANOVA is robust to this violation since the group with the largest sample size (salamanders) showed the most variance in multivariate dispersion for both bacteria and fungi (35). A significant effect of host (*F*_2, 132_ = 7.915, *R*^2^ = 0.096, *P* = 0.001), geography (*F*_7, 132_ = 3.258, *R*^2^ = 0.138, *P* = 0.001), and the interaction term (*F*_3, 132_ = 2.014, *R*^2^ = 0.037, *P* = 0.001) was observed for average bacterial assemblages. For fungi, a significant effect of host (*F*_2, 132_ = 9.989, *R*^2^ = 0.113, *P* = 0.003), geography (*F*_7, 132_ = 4.432, *R*^2^ = 0.175, *P* = 0.004), and the interaction term (*F*_3, 132_ = 2.406, *R*^2^ = 0.027, *P* = 0.001) was observed for average assemblages. These patterns were substantiated by the PCoA plots for both bacteria and fungi (Fig. 3) and reinforced that herptile gut microbiomes are shaped by host (e.g., reference 36), geography (e.g., reference 16), and their interactions.

### The herptile gut microbiomes harbor phylogenetically diverse *Basidiobolus* operational taxonomic units

**Fig 4:**
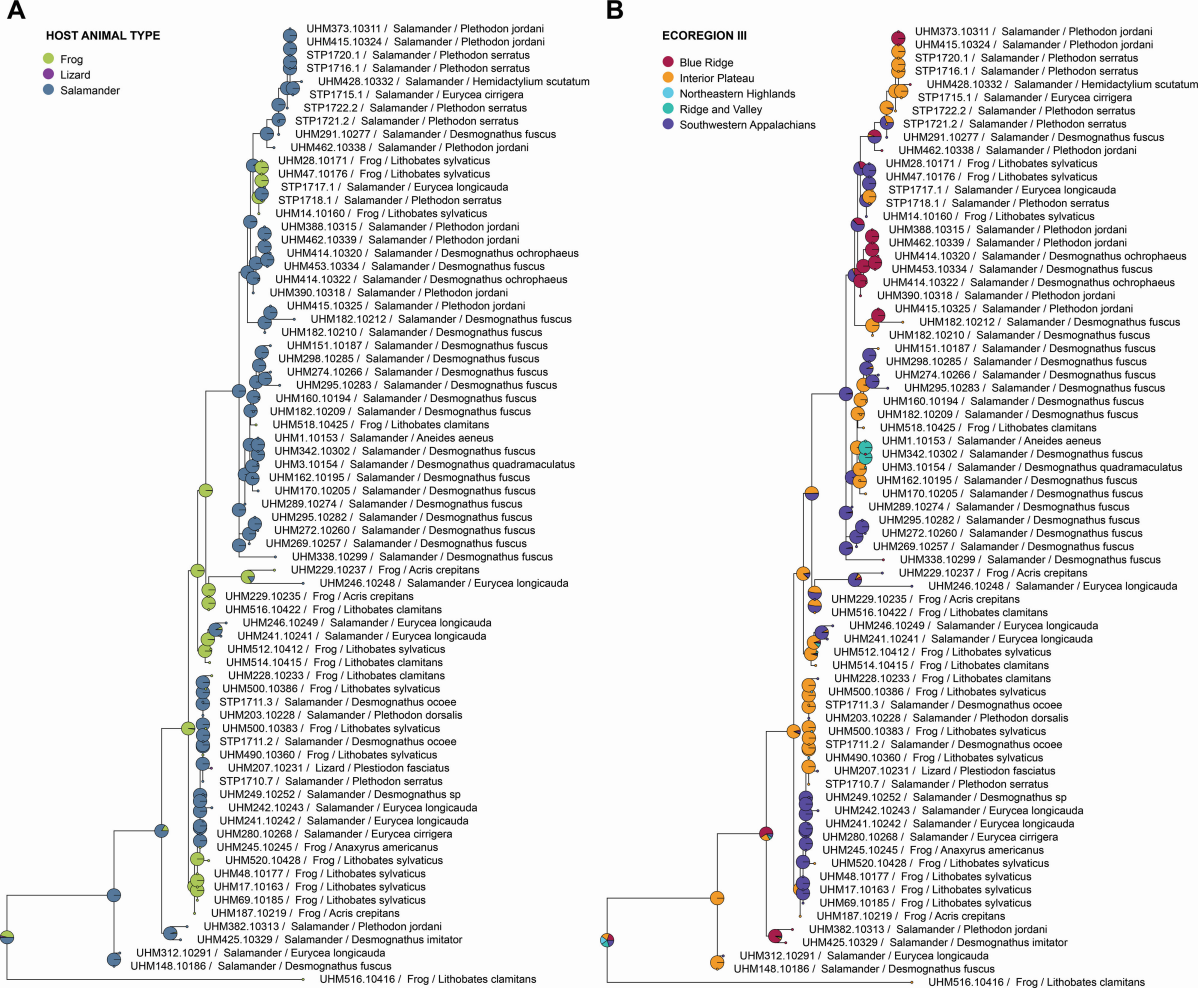
Ancestral character state reconstruction. Analysis testing host groups (**A**) and ecoregion III (**B**) classifications. Pie charts are indicative of the relative likelihoods of each node being in a particular state.

A total of 336 *Basidiobolus* ITS rDNA sequences were aligned and analyzed phylogenetically (Fig. S1). Fifty-two corresponded to reference sequences downloaded from NCBI, and the remaining 284 corresponded to living cultured isolates obtained in this project. Phylogenetic analysis revealed that *Basidiobolus* isolates represent nine well-supported clades (>87%, outlier of 54%). The tree is composed of two principal clades with strong bootstrap values (85%): one consisting of a group of 26 sequences archived on NCBI GenBank and three new *Basidiobolus* isolates collected in this project from a single frog (*Lithobates clamitans*) individual. A second clade was represented by 26 reference sequences and 281 study sequences (Fig. 4)*,* many of which were associated with one host species, with some showing geographic specificity at the EPA Ecoregion IV level (see colored bars, Fig. S1).

A single ITS sequence variant of *Basidiobolus* was collected from most herptile individuals using Sanger sequencing (*n* = 274 out of 284 individuals). But 10 individual amphibian hosts (*Eurycea longicauda* [*n* = 4], *Desmognathus imitator* [*n* = 1], *Desmognathus fuscus* [*n* = 1], *Desmognathus* sp. [*n* = 1], *Lithobates sylvaticus* [*n* = 1], and *L. clamitans* [*n* = 2]) were co-colonized by genetically different isolates of *Basidiobolus* (Fig. S1), demonstrating the ability of the herptile gut microbiome to simultaneously harbor multiple *Basidiobolus* operational taxonomic units (OTUs). Similar patterns of host-fungal specificity have been observed in the gut mycobiome of termites (37) and slimy salamanders (16).

To compare current and past work (16), we used comparisons of OTUs, amplicon sequence variants (ASVs) and phylogenetic analysis of Sanger sequence data. There are only 10 described species of *Basidiobolus*, but ITS rDNA data from amplicon metabarcoding studies that sampled herptile fecal samples are consistent with there being substantial undescribed biodiversity (Fig. S1). However, allelic variation in the *Basidiobolus* ITS rDNA marker complicates this interpretation. For example, from 59 slimy salamander fecal samples Walker et al. (16) found 485 *Basidiobolus* OTUs clustered at 97% similarity, and only two names could be provisionally linked to five of the OTUs with species epithets. Furthermore, only four species of *Basidiobolus* (*B. heterosporus*, *B. magnus*, *B. microsporus*, and *B. ranarum*) are found in the UNITE v.9.0 reference database (38). It is unlikely that all 485 OTUs were representatives of phylogenetic species, as we have found 4–14 ITS rDNA ASVs in the DNA of six living cultures of *Basidiobolus* (Table 1)*,* and *B. meristosporus* is estimated to have more than a thousand ITS rDNA copies in its genome (39). However, PCR and Sanger sequencing of genomic DNA isolated from cultures produced a single ITS product, which mapped to the dominant ASV detected in cultures via Illumina sequencing of amplicons (Fig. S2).

**TABLE 1:** Allelic diversity of ITS rDNA marker gene in living isolates of *Basidiobolus^a^*

Isolate ID	Fungal ID	Host ID	Total number of *Basidiobolus*ASVs	Abundant *Basidiobolus* ASVs (*n* > 5 reads)
STP1710.7	*Basidiobolus* sp.	*Eurycea longicauda* (long-tailed salamander)	13	12
STP1718.1	*Basidiobolus* sp.	*Plethodon serratus* (southern red-backed salamander)	4	4
STP1717.1	*Basidiobolus* sp.	*Plethodon serratus* (southern red-backed salamander)	6	4
STP1715.2	*Basidiobolus* sp.	*Eurycea cirrigera* (southern two-lined salamander)	14	12
UHM1.3285	*Basidiobolus* sp.	*Aneides aeneus* (green salamander)	6	6
UHM3.3284	*Basidiobolus* sp.	*Desmognathus quadramaculatus* (blackbelly salamander)	8	6

^*a*^ Data were generated using 2 × 250 bp paired end sequencing on an Illumina MiSeq and analyzed in DADA2.

The ancestral character state reconstruction (ACSR) analysis of frog, lizard, and salamander resolved a mixing of hosts across the ITS phylogeny (Fig. 4A). Salamanders were resolved as the dominant ancestral host of *Basidiobolus*, which may indicate an evolutionarily and/or ecologically meaningful interaction between host life history and the *Basidiobolus* life cycle, an interpretation consistent with a higher frequency of *Basidiobolus* detected in salamander fecal samples versus frogs and lizards (Fig. 2A). The ACSR analyses of geography also indicated a mixing of ecoregions suggesting that some OTUs, or closely related OTUs, are distributed across multiple ecoregions (e.g., ubiquitous species in the interior plateau and southwestern Appalachians) while others are more restricted in their distributions (e.g., Blue Ridge and Ridge and Valley; Fig. 4B).

Patristic distances from the ITS tree were used to test the differential effect of host and geography, and their interaction, across the *Basidiobolus* isolates sampled. Multivariate dispersion was not significantly different among host groups (betadisper; *F*_2, 70_ = 0.7065, *P* = 0.394), host genera (betadisper; *F*_8, 64_ = 1.8513, *P* = 0.102), or ecoregion III (betadisper; *F*_4, 68_ = 2.2075, *P* = 0.126). There was a significant effect of *Basidiobolus* genetic variation based on host group (frogs, lizards, and salamanders; *F*_2, 72_ = 3.6322, *R*^2^ = 0.0659, *P* = 0.016), ecoregion III (*F*_2, 72_ = 6.3010, *R*^2^ = 0.2286, *P* = 0.002) and the interaction term (*F*_2, 72_ = 6.8714, *R*^2^ = 0.1247, *P* = 0.020). A finer-scale assessment of host genus and the interaction term revealed non-significant effects (genus: *F*_*8*, 72_ = 2.2488, *R*^2^ = 0.1529, *P* = 0.086; genus × ecoregion III: *F*_8, 72_ = 3.2024, *R*^2^ = 0.1633, *P* = 0.070) on *Basidiobolus* genetic distance. While these results are consistent with a geographic effect and host:ecoregion interaction being more significant explanatory variables than host association, additional collections of *Basidiobolus* isolates from other hosts (e.g., lizards and frogs) will allow for more robust testing of hypotheses related to host association.

### *Basidiobolus* OTUs and bacterial gut communities are co-structured

Most research on herptile gut microbiomes has been descriptive in nature, focused entirely on bacteria, and with limited inference into microbial interactions (e.g., references 40, 41). Multiple factors (e.g., diet, host taxonomy, disease, and priority effects) are hypothesized to modulate gut bacterial assembly, though their relative contribution to this process has not been elucidated (42). For example, a study on ornamented pygmy frogs (*Microhyla fissipes*) revealed the complex remodeling of gut bacteria during metamorphosis and found a possible coevolution between gut microbial groups and host dietary shifts (43). Large-scale restructuring in the Burmese python (*Python bivittatus*) gut microbiome was observed to correspond with physiological changes in the host gut during snake feeding and fasting (44). The bacterial component of the herptile gut microbiome has been linked with diet (45), parasitic worm load (46), specific digestive system organs (36), and may exhibit metagenomic plasticity (47). A large-scale characterization of the gut bacterial microbiome of Mammalia, Aves, Reptilia, Amphibia, and Actinopterygii showed that diet determines specific functional guilds while host evolutionary history selects for prevalence of particular OTUs (42). Although we have a rudimentary understanding of herptile gut bacteria, no study to date has attempted to characterize the structure, function, and interactions between more than one domain of life composing natural herptile gut microbiomes.

**Fig 5:**
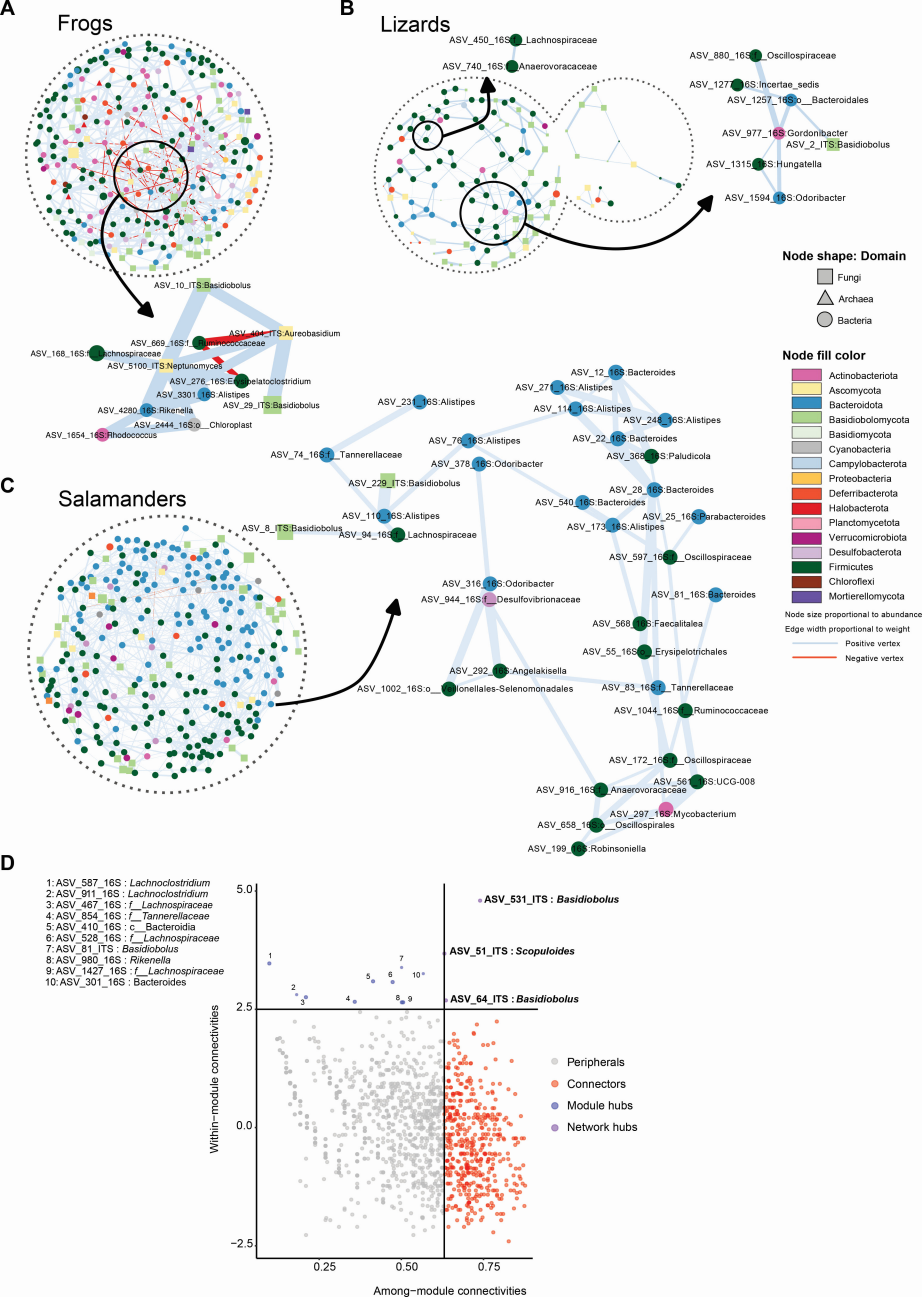
Co-occurrence network analysis of herptile host microbiomes including archaea, bacteria, and fungi. Networks from (**A**) frogs, (**B**) lizards, and (**C**) salamanders. Each node represents an ASV and is shaped according to the taxonomic domain. Edge color denotes a positive (blue) or negative (red) interaction between two connected ASVs with the width proportional to weight. Enlarged network subplots show the ASVs chosen based on highest degree of interaction and betweenness. (**D**) Scatter plot showing distribution of archaeal, bacterial, and fungal ASVs according to their within-module and among-module connectivity. Each dot represents an ASV in the complete data set of all herptile hosts. The four panels show the role distribution of selected groups of microbes. ASVs representing module hubs are indicated by numbers on the plot and listed in the upper left panel (1–10).

Microbial co-occurrence networks were constructed to explore associations within the gut of herptile hosts. Specific network variables were evaluated (for more detailed description of variables assessed in the network analysis, see Text S1). We found the overall structure of the microbial co-occurrence networks to be different between host animals (Fig. 5). The edge density represents how dense the network is in terms of edge connectivity and significance of associations. Frog gut microbiomes had the lowest edge density (0.0207) compared to salamanders (0.0257) and lizards (0.0261). The transitivity, or clustering coefficient, was the highest in frogs (0.1820) with their network containing 15 modules, followed by lizards (0.1704; 13 modules) and salamanders (0.1662; 10 modules). Higher clustering coefficient denotes the presence of communities or groups of nodes that are densely connected internally and forming modules. Modules in microbial co-occurrence networks provide insight into ecological processes within microbiomes that influence microbial community structure, such as niche filtering and habitat preference, between specific groups of microbes (48). Modules may also reflect the presence of functional and metabolic interactions between microbes that are potentially syntrophically coupled (49). Intensity of the within and between module connections is displayed through modularity of each network. The highest modularity was detected in lizards (0.781) compared to frogs (0.625) and salamanders (0.529). This demonstrates higher associations between specific microbes and may indicate the presence of stronger metabolic interactions shaping functional modules within the gut of lizards compared to frogs and salamanders. The frequency of positive associations between pairs of microbes was high in all three hosts with only 8% of negative associations (red edges, Fig. 5A) in frogs, 0% in lizards (Fig. 5B), and 0.46% in salamanders (Fig. 5C). Microbes might be co-present (or co-absent) across different animal hosts for various reasons; they may benefit from each other’s presence, have similar limitations for dispersal, or common niche requirements. These positive and negative associations can also be formed by higher-order interactions like competition, where microbes co-exist based on their differential ability to produce or tolerate toxins (50).

Centrality measures, including degree and betweenness, can indicate the importance of nodes in a network (51). To distinguish influential microbes in the microbial co-occurrence network, we made subnetworks of nodes with the top 30% highest degree of centrality scores (Fig. 5D). In frogs, an ASV of *Aureobasidium* (ASV-404-ITS; Ascomycota) had the highest betweenness scores, demonstrating a dominant role in the network. *Aureobasidium* showed negative association with ASV669-16S from the family Ruminococcaceae (phylum Bacillota) and a positive association with two *Basidiobolus* ASVs (ASV-10-ITS and ASV-29-ITS), *Neptunomyces* (ASV-5100-ITS; phylum Ascomycota), and *Alistipes* (ASV-3301-16S; phylum Bacteroidetes). Two high-scoring subnetworks were selected for lizards including positive associations between bacteria from the phylum Bacillota (ASV-450-16S: *Lachnospiracaea*, ASV-740-16S: *Anaerovoracaceae* dual node cluster and ASV-880-16S, ASV-1277-16S, and ASV-1315-16S: *Hungatella* main cluster), the Bacteroidetes (ASV-1257-16S and ASV-1597-16S: *Odoribacter*), the Actinomycetota (ASV-977-16S: *Gordonibacter*), and an ASV of *Basidiobolus* (ASV-2-ITS). For salamanders, the only fungal ASV selected with a dominant role occurred in the genus *Basidiobolus* (ASV-8-ITS and ASV-229-ITS). Overall, in all three host groups, fungal and bacterial co-occurrence networks detected strong and consistent interactions between nodes annotated as *Basidiobolus* and bacterial nodes belonging to Bacteroidota and Firmicutes.

To identify key functional groups in herptile gut microbiomes, nodes were classified into four categories of peripherals, connectors, module hubs, and network hubs (Fig. 5D) (52, 53). Peripheral ASVs can be interpreted as specialists, whereas module hubs and connectors are generalists, and network hubs are super-generalists (52). Connectors, generalists, and super-generalists are considered to be keystone microorganisms playing a critical role in network structure (54). Two ASVs of *Basidiobolus* and one *Scopuloides* (crust fungi belonging to Basidiomycota) were identified as network hubs (Fig. 5D). Nine ASVs belonging to Bacteroidetes and Firmicutes and one ASV of *Basidiobolus* comprised module hubs, denoting key roles of these microbes in the structure and stability of herptile gut microbiomes.

**Fig 6:**
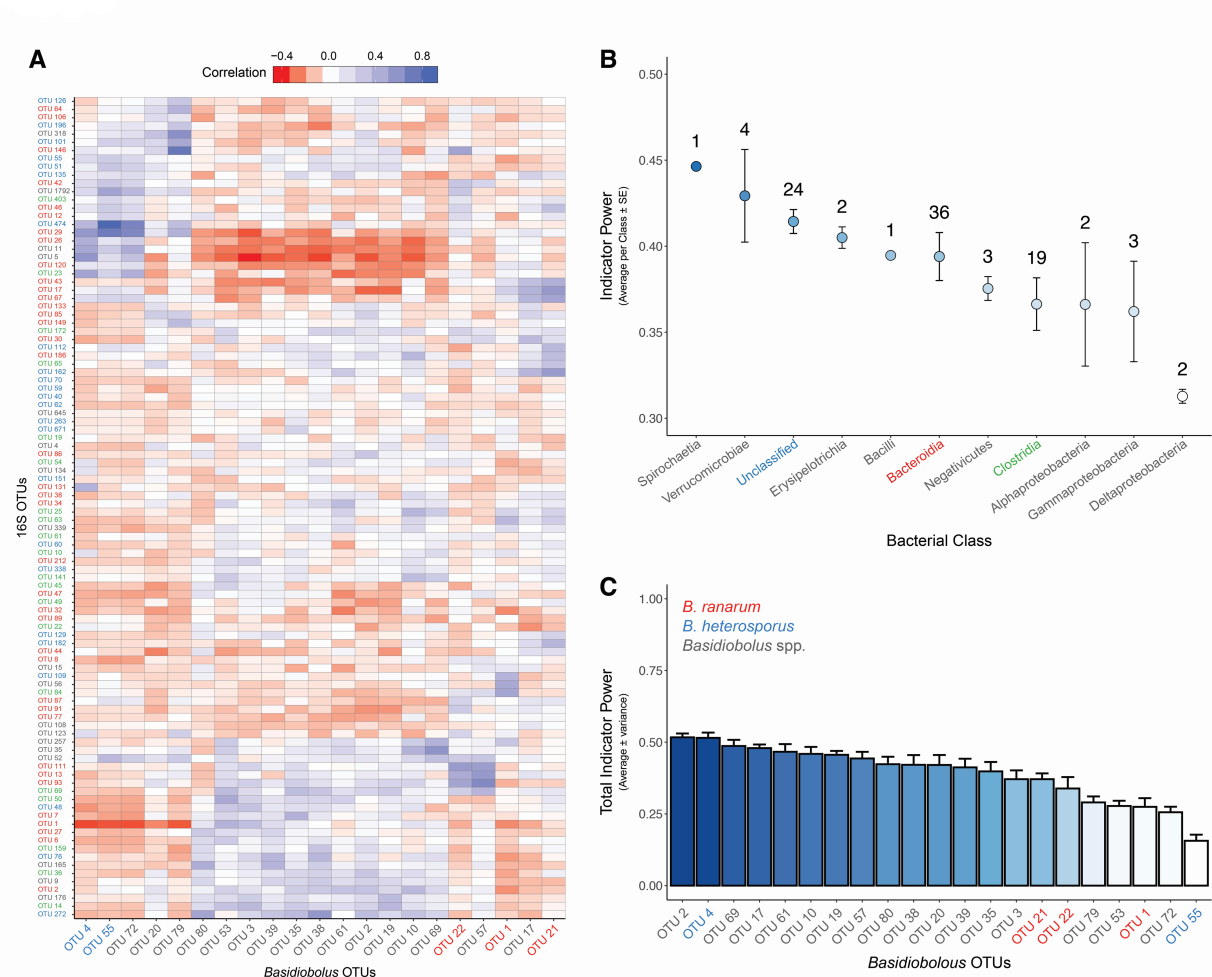
Indicator power analysis. (**A**) Heatmap of the Pearson correlation coefficients between the most abundant bacterial OTUs (*n* = 100) and abundant *Basidiobolus* OTUs (reads ≥10; *n* = 21 OTUs). Cooler heatmap colors indicate that a bacterial OTU is positively correlated with a *Basidiobolus* OTU. Conversely, warmer heatmap colors indicate that a bacterial OTU is negatively correlated with a *Basidiobolus* OTU. Hierarchical clustering was performed on the correlation matrix using the complete linkage algorithm. The clustering methodology was used to arrange the *Basidiobolus* OTUs on the *x*-axis and bacterial OTUs on the *y*-axis (colored text corresponds to bacterial classes in panel B). (**B**) Using the 100 most abundant bacterial OTUs, mean indicator power was calculated for each bacterial class. This value represents the ability of an OTU to predict the occurrence of all *Basidiobolus* OTUs present in the data set (blue to white color gradient is a proxy for the strength of the indicator power). OTUs from bacterial classes with high mean indicator power are strong predictors of the presence or absence of *Basidiobolus*. The number of OTUs from each bacterial class are annotated above each point. (**C**) Total indicator power, a measure of the ability of one *Basidiobolus* OTU to predict a complete assemblage of bacterial OTUs, was calculated for each *Basidiobolus* OTU. *Basidiobolus* OTUs with low total indicator power values (e.g., OTU55) are not well correlated with the overall structure of the herptile gut microbiome. *Basidiobolus* OTU labels classified using the UNITE as belonging to the same species are assigned distinct colors: red*—B. ranarum*, blue*—B. heterosporus*, and gray*—Basidiobolus* spp.

Walker et al. (16) determined that species in the genus *Basidiobolus* averaged 60% (minimum 8.1%; maximum 97.8%) of the relative abundance of all gut fungi among individuals in the slimy salamander species complex. We demonstrate here that bacterial OTUs in the slimy salamander gut microbiome were correlated with the relative abundance of *Basidiobolus* OTUs (Fig. 6A). *Basidiobolus* OTUs classified as belonging to the same species are predicted by similar bacterial OTUs and clustered nearer to one another (Fig. 6A). Several unidentified species of *Basidiobolus* were also determined to correlate with at least 10 classes of bacteria including the Verrucomicrobiae, Bacteroidia, and Clostridia (Fig. 6B). Mean indicator power values (55) of each bacterial class were used to determine if there was taxonomic variation in the ability of bacterial OTUs to predict *Basidiobolus* occurrence in slimy salamander gut microbiomes. A single OTU in the class Spirochaetia was the strongest indicator (IP = 0.446) of *Basidiobolus* (Fig. 6B). Numerous OTUs in the Bacteroidia (*n* = 36) and the Clostridia (*n* = 19) were weaker, but more abundant indicators of *Basidiobolus*. We examined whether the occurrence of certain *Basidiobolus* OTUs was correlated with the assemblage of bacterial OTUs using total indicator power (TIP; Fig. 6C). Results suggest that *Basidiobolus* OTUs do not interact equally with bacterial assemblages in the slimy salamander gut microbiome. *B. ranarum* OTUs had higher TIP values than *B. heterosporus* (Fig. 6C) suggesting that different species of *Basidiobolus* display variability in the strength of their interactions with gut bacteria.

### Horizontal gene transfer and its connection to specialized metabolism in *Basidiobolus*

The best-documented case of HGT in fungi involves the AGF with an identified HGT rate of 2.0–3.5% (30). Phylogenomic analyses of three draft *Basidiobolus* genomes reveal a similar magnitude of HGT as AGF, however, with 2–5% of genes predicted to have bacterial origins, with the largest sources being Actinobacteria, Firmicutes, and Proteobacteria (32). The most pronounced signal of HGT is in secondary or specialized metabolism for nonribosomal peptide synthetases, which are known to function in immunoregulation, quorum sensing, iron metabolism, and siderophore activity (NRPS; Fig. S3). It seems plausible that the herptile gut environment promotes HGT from bacteria to fungi under the selection pressure of acquisition of novel metabolism necessary to adapt to herptile microbiomes.

**Fig 7:**
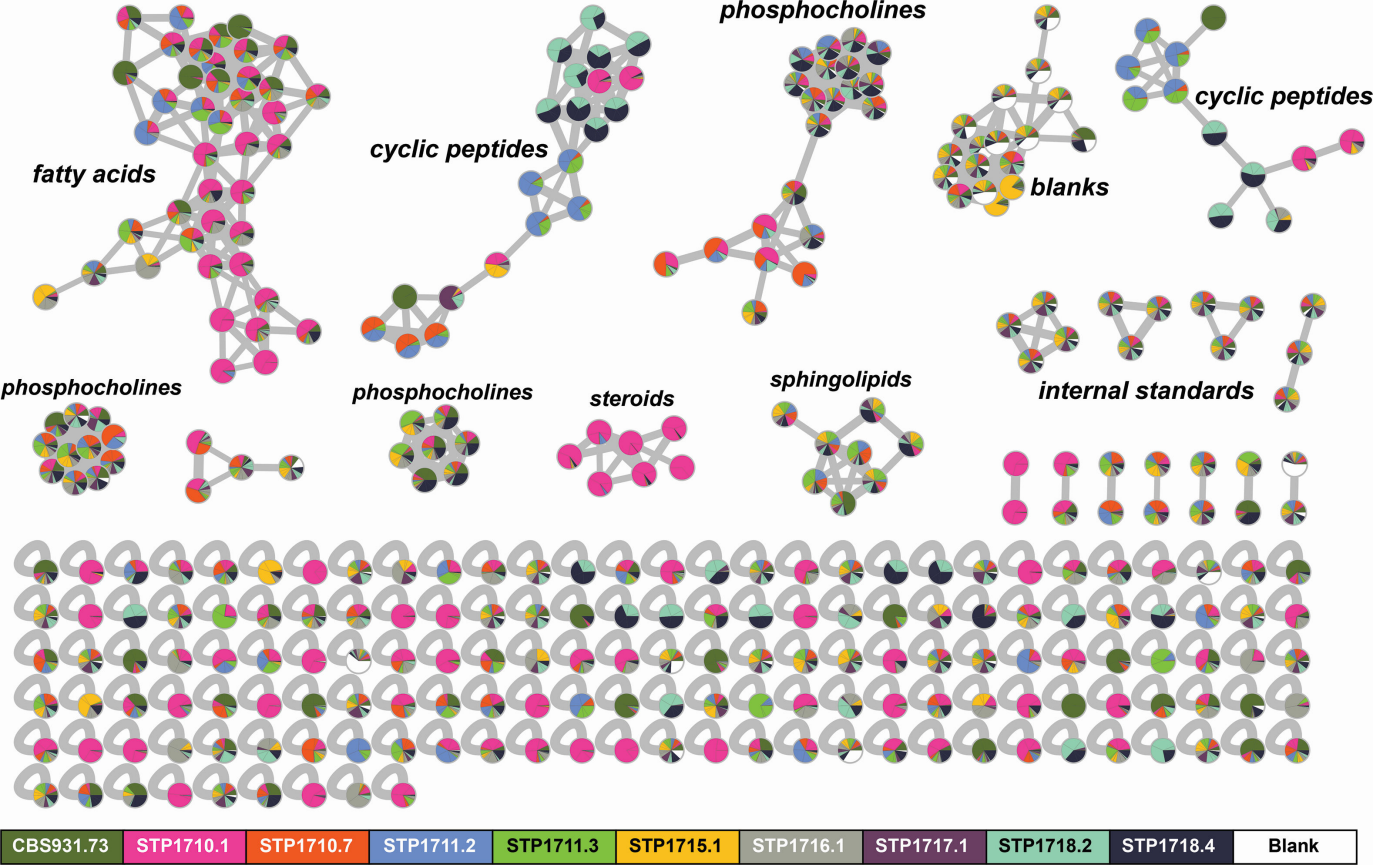
GNPS feature-based molecular network of untargeted LC-MS/MS data for extracts of 10 different *Basidiobolus* isolates cultured on potato dextrose agar. Mass features are represented as nodes and are colored according to source isolate, with pie charts representing mass features shared between *Basidiobolus* isolates. Edges connect mass features (nodes) with similar MS/MS spectra, defined as a cosine similarity score >0.7, which indicates structural relatedness. *Basidiobolus* isolates derived from feces of a gecko (*B. meristosporus* CBS 931.73) and salamanders *Eurycea longicauda* (STP1710.1 and STP1710.7, one animal), *Desmognathus ocoee* (STP1711.2 and STP1711.3, one animal), *Eurycea cirrigera* (STP1715.1, one animal), and *Plethodon serratus* (STP1716.1, STP1717.1, STP1718.2, and STP1718.4, three animals). General structural class was determined by manual analysis of GNPS library hits and outputs from Sirius 5.6.3 and CANOPUS for multiple nodes within a subnetwork.

To test for the production of metabolites consistent with NRPS biosynthesis, we cultured nine *Basidiobolus* isolates from six salamander individuals, as well as *B. meristosporus* CBS 931.73, in parallel on potato dextrose agar (PDA) for LC-MS/MS profiling. Each *Basidiobolus* culture plate produced 150–500 mg of dried mycelium and resulted in an average of 4.2 mg of extract (average of 18.7 mg of extract per gram of dried mycelium). Data processing in MzMine resulted in selection of 331 mass features (*m*/*z* and retention time) with associated quantification (area under analyte chromatographic peak/area under internal standard chromatographic peak) across all samples. Feature-based molecular networking (56) of the resulting MS/MS spectra using the GNPS online platform (57) yielded nodes and subnetworks containing mass features from all 10 *Basidiobolus* cultures (Fig. 7). The GNPS feature-based molecular network assigned 613 edges between the 331 nodes and spectral library annotations for 21 of those nodes. General chemical class assignments for larger sub-networks were determined by comparison of GNPS annotations (when applicable) with both SIRIUS and CANOPUS outputs for many of the networked nodes. While the identity of each node cannot be determined at this level of analysis, a general overview shows one large subnetwork containing fatty acids, three sub-networks of phosphocholines, two cyclic peptide sub-networks, one containing steroids, and one with sphingolipids. Excluding the steroid subnetwork (pink), primarily derived from STP1710.1, the remaining chemical classes are generally shared between all *Basidiobolus* isolates. For example, both cyclic peptide subnetworks contain nodes representing mass features present in more than one of the isolates. Nodes with contributions from different *Basidiobolus* isolates in the two cyclic peptide subnetworks are consistent with a conservation of biosynthetic potential for these specialized metabolites at the genus level. Notably, in both cyclic peptide subnetworks, *Basidiobolus* isolates from the same animal share the same structurally related mass features (e.g., STP1718.2 and STP1718.4) that are also linked to other structurally related mass features (nodes) found in extracts of other *Basidiobolus* isolates. In some cases, *Basidiobolus* isolates from different animals of the same species share some cyclic peptide mass features, for example, STP1717.1 and STP1718.2 from two different southern red-backed salamander (*Plethodon serratus*) individuals. The conservation of multiple specific cyclic peptide mass features between *Basidiobolus* isolates from different salamander species is also striking. For example, *Basidiobolus* isolate STP1710.7, from a long-tailed salamander (*E. longicauda*), and STP1711.2 and STP1711.3 isolates from the same Ocoee salamander (*Desmognathus ocoee*) share multiple nodes in both cyclic peptide subnetworks.

A number of investigations of the skin microbiomes of frogs and other herptiles have identified specialized bacterial metabolites, for example, prodigiosin, violacein, and volatile metabolites, such as antifungals against *Batrachochytrium* pathogens (58). In contrast, there appear to be no untargeted metabolomics studies of herptile gut microbiomes, and only two separate reports of specialized metabolites from cultured bacteria isolated from herptile guts. The latter publications document the antibacterial activity of conditioned media filtrates from cultured gut bacteria and LCMS-based annotations of filtrate metabolites for a water monitor lizard (59) and a turtle (60). Subsequent antibacterial testing of commercially available metabolites annotated from the turtle-derived bacteria was also reported (61).

To our knowledge, antifungal basidiosins A–L (62, 63, 64) and meristosporins A–C (64) are the only published specialized metabolites isolated and characterized from laboratory-cultured *Basidiobolus* isolates (62). These structurally similar cyclic pentapeptides from *B. meristosporus* (isolate ARSEF 4516) contain both D and L amino acids. MK3990 (CAS #136509-32-5) is another peptidic metabolite referenced in a 1991 Japanese patent application as an antibiotic produced by *B. meristosporus*, although no molecular structure is directly available. Notably, *Basidiobolus* genomes are enriched in specialized metabolite biosynthetic genes compared to other related fungi (32), and particularly in NRPS genes. Indeed, we have observed several peptidic metabolite signatures in untargeted mass spectrometry experiments and these metabolites are shared across strains of *Basidiobolus* isolated from different hosts, consistent with their genomic enrichment for NRPSs as a characteristic of the genus.

### Conclusion

Herptile gut microbiomes are well suited to test and refine hypotheses regarding fungal adaptations to the animal gut microbiome environment and microbial interactions between filamentous fungi and bacteria. *Basidiobolus* is a common and abundant member of herptile gut microbiomes, and there appears to be a large amount of undescribed diversity in this genus. The bacterial communities of these systems are significantly structured by host and geography, but they are also correlated with the presence and absence of *Basidiobolus*, consistent with fungal-bacterial interactions. Like the anaerobic gut fungi of ruminant mammals, we propose that the herptile gut microbiome promotes HGT from bacteria to *Basidiobolus*. In doing so, it selects for genes that lead to fungal adaptation to the herptile gut environment and for specific metabolic traits that function in natural microbiomes. Genomic and chemical analyses were consistent with *Basidiobolus* being uniquely equipped to synthesize a diversity of peptidic metabolites, for which the core biosynthetic genes are hypothesized to be bacterial in origin. An interdisciplinary approach comprising ecology and evolutionary biology, genomics and metagenomics, natural product chemistry, and experimental biology is required to advance new scientific understanding of the mechanisms involved in interkingdom microbial interactions and the structuring of these natural gut microbiome systems.

## MATERIALS AND METHODS

### Collection of animals and processing fecal samples

Animals included in this study were collected between 2014 and 2022. Collection information including host taxonomy and geographic location of EPA ecoregion III and IV levels are included in Table S1. Details of animal collection and collection of fecal samples are provided in Walker et al. (16), but briefly: animals were collected into plastic bags with a small amount of field material (e.g., leaf litter). Each animal was given a unique collection number, and the collection site was flagged with the same collection number. Animals were transported to processing sites (e.g., field labs), where they were removed from the field collection bags, placed in a new plastic bag, and surfaced washed with sterile ddH_2_0 for approximately 1 min to remove debris and transient microbes. After washing an animal, a skin swab and a tail or toe clip was obtained, then the animal was placed in a moist chamber overnight. In the morning, fecal samples were collected with a sterile plastic scoopula. For animals collected from 2014 to 2018, fecal samples were placed into empty sterile microcentrifuge tubes, frozen and stored at −80°C until being thawed, diluted in 1 mL sterile molecular grade water, and processed using the protocol below. For animals sampled in 2022, fecal samples were placed into 1 mL of sterile molecular grade water, then vortexed for 20 s and aliquoted as follows: 100 µL in 20% glycerol for culturing bacteria, 250 µL for DNA extractions, 250 µL for culturing *Basidiobolus*, and 400 µL for chemical analyses. Animals were then returned to their respective collection site after collection of fecal samples. A total of 33 different species were sampled from 16 frogs, 90 salamanders, and 35 lizards (Table S1).

### Culturing, microscopy, and imaging

Isolation of *Basidiobolus* was attempted for all 207 animals that produced fecal pellets in the 2022 field season. Canopy plates (65, 66) were prepared as follows: five ~50 µL drops from the 250 µL fecal sample aliquot mentioned above were applied to the paper towel surface of the canopy plate. The plates were incubated with the paper towel (lid) surface side down with desk lamp illumination at ambient room temperature. Plates were monitored for *Basidiobolus* spore discharge over 2–5 days, after which plates were autoclaved. Forcibly discharged blastoconidia with germinating hyphae were isolated from the PDA surface using sterile dissecting needles and stereoscope at ~50× total magnification onto PDA plates.

Random selection of *Basidiobolus* isolates were grown in full-strength PDA and corn meal agar (CMA) for 1 week at 25°C. Fresh *Basidiobolus* cultures were used to prepare slide cultures (67) which were incubated at 25°C until the mycelia were observed on the cover slip. Coverslips were mounted on slides and stained with lactophenol cotton blue solution (Sigma) and observed under the light microscope. Microscope images were captured with a Leica DMC 4500 camera, using the Leica Application Suite v4.12.

### Amplicon sequencing and analysis—16S rRNA and ITS rDNA markers

Target-gene data collection resulted in three data sets: (i) a broad sampling of 33 species of reptiles and amphibians that included 16 frogs, 90 salamanders, and 35 lizard individuals from the midwestern, southeastern, and southwestern United States; (ii) a selection of six *Basidiobolus* living strains isolated from different salamander species; and (iii) a focused sampling of 60 slimy salamanders, which is a group of congeneric species of *Plethodon* that have only recently diverged and still hybridize, from 13 sites in the southeastern United States (Table S1).

DNA was extracted from fecal pellets using the Qiagen DNeasy 96 PowerSoil Pro kit and high-throughput sequencing was completed on an Illumina MiSeq (2 × 250 bp paired-end) for the 16S rRNA V4 and ITS1 rDNA markers as in Walker et al. (16). For bioinformatic analyses, primers of the 16S-V4 gene were removed from forward and reverse raw reads with Cutadapt v4.1 (68). Reads were filtered, dereplicated, trimmed (forward reads to 230 bp and reverse reads to 160 bp), and merged (min overlap of 100 bp) with R package DADA2 v1.24.0 (69). After inference of the ASVs, chimeric sequences were removed, and taxonomy was assigned to each ASV using the naive Bayesian classifier method against the SILVA v.138 reference taxonomic database (70). ITS1 rDNA reads were first extracted from raw reads with ITSxpress v1.7.2 (71) and merged with BBMerge (72). Reads were filtered (min length of 50 bp) and ASVs were inferred with DADA2 v1.24.0 (69). After the removal of chimeric sequences, taxonomy was assigned to each ASV using the naive Bayesian classifier method against the UNITE + INSD fasta release v8.3 (73). For all data sets, the function isContaminant from the R package Decontam v1.16 (74) was used to identify and discard contaminant ASVs. Contaminants were identified using the option “method = frequency” which selects ASVs whose relative abundance varies inversely with sample DNA concentration. Decontamination was run at a range of probability threshold values (from 0.05 to 0.95, increasing by 0.10). This is the probability threshold below which the null hypothesis (that an ASV is not a contaminant) should be rejected. For each data set, we determined what percentage of sequences were removed from no-template control (NTC) libraries relative to sample libraries. A threshold value was selected if it was the last value at which more sequences were removed from NTC than sample libraries relative to the next value tested (given at least 10% removal of total NTC sequences). Both data sets were then rarified to a depth of 10,000 reads using the function rrarefy from the R package vegan v2.6-4 (75). To understand intragenomic ITS rDNA allelic diversity, ITS rDNA amplicon sequencing was performed on six living isolates of *Basidiobolus*. Sequencing was performed as described in Walker et al. (16); however, the bioinformatic analysis was completed as described above in DADA2.

The fungal ITS rDNA and 16S rRNA marker data sets were analyzed with R package Phyloseq v1.40.0 (76). The 20 most abundant fungal and bacterial genera were visualized in bar charts, highlighting differences among hosts and geography (states) (Fig. 2). Beta diversity of both data sets was inspected with a principal coordinate analysis (PCoA) based on Bray-Curtis distances. A Betadisper analysis was performed to test for differences in multivariate dispersion between host groups. PERMANOVA tests (function adonis) were used to compare average microbiome assemblages among animal hosts, across geographic locations and the interaction between host and geography (R package Vegan v2.6.4) (75). Relative abundances of bacterial and fungal taxa among the different animal hosts were visualized with R package ampvis2 v2.7.34 (77).

### Sanger sequencing, phylogenetic reconstruction, and ancestral state reconstruction of *Basidiobolus*

Genomic DNA was extracted from living isolates using Extract-N-Amp method and the amplification of the ITS region from rDNA was performed with the primers ITS5 (5′-GGAAGTAAAAGTCGTAACAAGG-3′) and ITS4 (5′-TCCTCCGCTTATTGATATGC-3′) (78). The thermal cycling conditions were as follows: an initial denaturation step at 95°C for 2 min, followed by 30 cycles of 95°C for 30 s, 55°C for 1 min, and 72°C for 1 min, and a final extension step at 72°C for 5 min. PCR reactions (25 µL final volume) contained 2 µL of genomic DNA, 1.25 µL of each primer (10 µM each), 0.5 µL nucleotide mix (10 mM), 2.5 µL MgCl_2_ (25 mM), 5 µL GoTaq Reaction Buffer (5×), 0.25 µL GoTaq DNA Polymerase (5 U/µL; Promega, Radnor, PA, USA), and 12.25 µL of molecular grade water. PCR products were cleaned with ExoSap-IT (Thermo Fisher Scientific, Waltham, MA, USA) and sequenced at the Center for Quantitative Life Sciences (Corvallis, OR, USA).

A total of 52 sequences from different species of *Basidiobolus* were downloaded from the NCBI GenBank database and aligned with the ITS sequences of *Basidiobolus* isolates. An ITS rDNA alignment was constructed using MAFFT v7 (79) and visualized and edited in Geneious Prime v2022.2 (https://www.geneious.com). A maximum likelihood phylogenetic tree was constructed with RAxML-HPC v8.0 (80) under GTR + GAMMA + I model with 1,000 bootstrap replicates (81). The final tree was visualized in FigTree v1.4.4 (http://tree.bio.ed.ac.uk/software/figtree/).

An ACSR analysis was performed on a selected group of *Basidiobolus* ITS sequences representing different hosts to investigate the association of different herptile hosts and geographic location (ecoregion level III) at specific nodes. To perform this analysis, all ITS sequences used to construct the phylogenetic tree (Fig. S1) were clustered into OTUs at 99% similarity using VSEARCH v2.22.1 (82). To maintain representation of hosts for each OTU, an ITS sequence was included from each *Basidiobolus* isolated from a unique host genus within each 99% OTU, resulting in seventy-three sequences. These *Basidiobolus* ITS sequences were aligned, and a phylogenetic tree was constructed as previously described. Using FigTree, the phylogenetic tree was exported in nexus format for an ACSR analysis using the R Phytools package v1.2-0 (83). The results of ACSR analysis on host type and geographic location were displayed in two phylogenetic trees.

The phylogenetic tree constructed with the selected ITS sequences, was imported into Geneious v2023.0.4 (https://www.geneious.com), and a patristic distance matrix was calculated. The patristic distance matrix was used to test the effect of host groups, host genera and geographic location on the genetic change represented in the ACSR phylogenetic tree. A Betadisper analysis was used to test differences in multivariate dispersion between host groups. PERMANOVA (function adonis) was used to test the effect on the phylogenetic change among hosts groups (salamanders, frogs, and lizards), host genera, across geographic locations (Ecoregion III), and all interactions (R package Vegan v2.6.4) (75).

### Correlation between *Basidiobolus* and bacterial communities

A co-occurrence network analysis was performed on the complete data set of frogs, lizards, and salamanders with a frequency threshold of ASVs present in more than 20% of samples. After Clr transformation, the sparse inverse covariance estimation and model selection were implemented using the spiec.easi function in SPIEC-EASI with MB neighborhood selection (84). The nlambda was set for each model to obtain at least 0.49 or the closest possible value to the target stability threshold of 0.05. Data processing and networks construction were performed using R (version 4.2.2) (85); in the packages phyloseq v1.42.0 (76), SpiecEasi v1.1.2 (84), igraph v1.3.5 (86), and microbiomeutilities v1.00.17 (87) and their dependencies. Network properties including clustering coefficient, edge density, connectivity and betweenness were calculated in igraph and cytoscape. The module/submodule detection and modularity analyses were performed using fast greedy modularity optimization as a function in igraph (88). Networks were visualized in Cytoscape v3.9.1 (89) using the edge-weighted spring-embedded layout. We removed ASVs with <10 reads and 0.5% minimum abundance threshold to identify keystone bacterial or fungal ASVs in herptile gut microbiomes. The keystone microorganisms were identified by two parameters of within-module connectivity and among-module connectivity (52, 53).

Amplicon data from Walker et al. (16) including the 16S rRNA and ITS1 rDNA were analyzed as 97% OTUs for 60 slimy salamander fecal samples. These data were utilized to explore the extent of correlation between bacterial taxa and *Basidiobolus* OTUs in a congeneric host (*Plethodon* spp.) (16). Rare bacterial and *Basidiobolus* OTUs (<10 observations in data set) were removed prior to downstream analyses. An indicator power analysis (55) was used to determine the ability of the 100 most abundant bacterial OTUs to predict the presence/absence of the most abundant *Basidiobolus* fungal OTUs (reads ≥10; *n* = 21 OTUs). The average indicator power value was calculated for each bacterial OTU. These values were then grouped by bacterial class to determine if there was taxonomic variation in the ability of bacterial OTUs to predict *Basidiobolus* occurrence. TIP, the average ability of the members of an indicator assemblage to predict the occurrence of a target taxon, was calculated for each *Basidiobolus* OTU.

### Molecular network analyses

Ten different *Basidiobolus* isolates were grown over cellophane on potato dextrose agar for 21 days. Fungal mycelium was then collected and freeze-dried before the addition of HPLC grade MeOH (0.25 g of mycelium/mL). Suspensions were then sonicated for 30 min and left overnight. The extract was then filtered to remove mycelium and concentrated under reduced pressure. The extraction procedure and following analysis were also performed on a blank sample (empty vial) as a control. For tandem mass spectrometry analysis, fungal extracts were re-dissolved in LCMS grade MeOH (1 mg/mL) spiked with two internal standards (D-Ala2-odoamide [90], *m*/*z* 856.5474, 0.005 mg/mL; Tsn-Pc-832A [91], *m*/*z* 832.5404, 0.005 mg/mL). Full (0.5 mg/mL) and half (0.25 mg/mL) strength quality control samples containing six randomly chosen extracts were run at the beginning and end of the batch, and samples were run in random order, with a blank run every 10 samples. For each chromatographic run, 3 µL of sample was injected on an Agilent 1260 infinity II LC coupled to a 6545 QToF MS. For the chromatographic separation, a reversed-phase C18 porous core column (Kinetex C18, 50 × 2.1 mm^2^, 2.6 µm particle size, 100 Å pore size, Phenomenex, Torrance, USA) was used. The mobile phase consisted of solvent A (H_2_O + 0.1% formic acid [FA]) and solvent B (acetonitrile [ACN] + 0.1% FA), and the flow rate was 0.4 mL/min. After injection, the samples were eluted with a linear gradient from 0 to 0.5 min at 25% B, 0.5–7 min at 25–95% B, 7–8 min at 95% B, followed by a 3.5-min washout phase at 100% B and a 5-min re-equilibration phase at 25% B. The column compartment was maintained at 30°C. Data-dependent acquisition of MS^2^ spectra was performed in positive mode. Electrospray ionization parameters were set to a gas temperature of 325°C, a gas flow of 10 L/min, a nebulizer 20 psi, a sheath gas temperature of 375°C, and a sheath gas flow of 12 L/min. The spray voltage was set to 600 V. MS scan range was set to *m*/*z* 100–3,000 and the scan rate was 10 spectra/s. Collision energy was set to a stepwise increase from 20 to 40 to 60 eV. MS^2^ scans were selected when precursor counts reached 1,000 counts and spectra were excluded after six were collected. For MS^2^ data analysis, raw spectra were converted to .mzML files using MSconvert (ProteoWizard). MS^1^ and MS^2^ feature extraction was performed using MZmine2.53. Each feature ID represents a *m*/*z*-retention time pair and has an associated MS^2^ spectrum and quantification across samples based on area under the chromatographic peak. The parameters used in MZmine2.53 are listed in Table S2. The feature table .csv and .mgf files were exported and uploaded to GNPS (gnps.ucsd.edu) (57) for feature-based molecular networking (FBMN) (56). For spectrum library matching and spectral networking, the minimum cosine score to define spectral similarity was set to 0.7. The precursor and fragment ion mass tolerances were set to 0.02 Da, minimum matched fragment ions to 6. Molecular networks were visualized with Cytoscape 3.9.1 (89) and node information was enriched with the MS1 peak areas from the feature table.

Input .mgf files for Sirius 5.6.3 were exported from MzMine and opened in the Sirius 5.6.3 GUI (92). Jobs were run and filtered by an isotope pattern with an MS^2^ mass accuracy of 10 ppm. MS^2^ isotope scorer was ignored, and 10 candidates were stored for each mass feature. Possible ionizations included [M + H]^+^, [M + Na]^+^, and [M + K]^+^. ZODIAC (93) and CANOPUS (92, 94, 95) jobs were included with preset parameters. Masses greater than 850 Da were excluded.

## Data Availability

Raw ITS sequences from isolates, raw ITS and 16S amplicon sequence reads have been deposited in the NCBI/SRA database under the project accession PRJNA932855. Scripts and input data used for the analysis are available via a GitHub repository (https://github.com/herptilemicrobiomes/HerptileGutMicrobiome_2023). The link to the GNPS job is as follows: https://gnps.ucsd.edu/ProteoSAFe/status.jsp?task=ab180f58f4ee484683b7950461b22566.

## SUPPLEMENTAL MATERIAL

Text S1 msphere.00475-23-s0001.docx Definitions of terms used to describe network analyses.

Fig. S1 msphere.00475-23-s0002.tif ITS rDNA phylogeny of *Basidiobolus* species.

Fig. S2 msphere.00475-23-s0003.tif *Basidiobolus* ITS diversity and abundance.

Fig. S3 msphere.00475-23-s0004.tif Phylogenetic tree and abundance of NRPS adenylation domains (A-domains).

Tables S1 and S2 msphere.00475-23-s0005.docx Herptile species processed and pre-processing settings for mass feature detection using MZmine2.

Table S3 msphere.00475-23-s0006.xlsx Descriptive statistics of three hosts co-occurrence network analysis.

ASM does not own the copyrights to Supplemental Material that may be linked to, or accessed through, an article. The authors have granted ASM a non-exclusive, world-wide license to publish the Supplemental Material files. Please contact the corresponding author directly for reuse.
