# Supplementary material for: Herptile gut microbiomes: a natural system to study multi-kingdom interactions between filamentous fungi and bacteria
Source: mSphere. 2024 Feb 13;9(3):e00475-23. doi: 10.1128/msphere.00475-23 (PMC10964425; doi:10.1128/msphere.00475-23)
Supplement: Text S1 — Definitions of terms used to describe network analyses. [file msphere.00475-23-s0001.docx]

**Text S1. Definitions of terms used to describe network analyses (Fig. 5)**

1. **Modularity:** Modularity is the tendency for networks to consist of highly interconnected sub-groups of ASV nodes that are distinguished from other such groups or modules, by relatively sparse among-group connections (1).
2. **Degree:** the degree of a node equals the number of edges it has.
3. **Edge density:** The density of a graph is the ratio of the actual number of edges (cooccurrences) to the largest possible number of edges in the graph, assuming that no multi-edges are present.
4. **Betweenness centrality:** the betweenness centrality of a node is equal to the number of shortest paths between any two nodes in the graph passing through that node.
5. **Closeness centrality**: the closeness centrality of a node is given by the average distance of the node to any other node.
6. **Transitivity or clustering coefficient**: The clustering coefficient calculates the fraction of observed vs. possible triangles (clusters) of nodes.
7. For all given values the mean value is calculated from all nodes in the network.

**REFERENCE**

1. Clauset A, Newman MEJ, Moore C. 2004. Finding community structure in very large networks. Phys Rev E 70:066111.
