# Supplementary material for: Herptile gut microbiomes: a natural system to study multi-kingdom interactions between filamentous fungi and bacteria
Source: mSphere. 2024 Feb 13;9(3):e00475-23. doi: 10.1128/msphere.00475-23 (PMC10964425; doi:10.1128/msphere.00475-23)
Supplement: Tables S1 and S2 — Herptile species processed and pre-processing settings for mass feature detection using MZmine2. [file msphere.00475-23-s0005.docx]

| **Urodela (salamanders)** | | | **90** |
| --- | --- | --- | --- |
| *Ambystoma* | 7 | *Ambystoma jeffersonianum* | 5 |
|  |  | *Ambystoma maculatum* | 1 |
|  |  | *Ambystoma opacum* | 1 |
| *Desmognathus* | 7 | *Desmognathus aeneus* | 1 |
|  |  | *Desmognathus brimleyorum* | 5 |
|  |  | *Desmognathus welteri* | 1 |
| *Eurycea* | 1 | *Eurycea bislineata* | 1 |
| *Hemidactylium* | 5 | *Hemidactylium scutatum* | 5 |
| *Plethodon* | 69 | *Plethodon aureolus* | 6 |
|  |  | *Plethodon chattahoochee* | 10 |
|  |  | *Plethodon chattahoochee x chlorobryonis* | 1 |
|  |  | *Plethodon chlorobryonis* | 5 |
|  |  | *Plethodon glutinosus* | 5 |
|  |  | *Plethodon grobmani* | 4 |
|  |  | *Plethodon metcalfi* | 5 |
|  |  | *Plethodon mississippi* | 6 |
|  |  | *Plethodon montanus* | 5 |
|  |  | *Plethodon shermani* | 9 |
|  |  | *Plethodon teyahalee* | 13 |
| *Pseudotriton* | 1 | *Pseudotriton ruber* | 1 |
|  | | |  |
| **Anura (frogs)** | | | **16** |
| *Acris* | 10 | *Acris crepitans* | 10 |
| *Anaxyrus* | 1 | *Anaxyrus fowleri* | 1 |
| *Lithobates* | 5 | *Lithobates catesbeiana* | 2 |
|  |  | *Lithobates clamitans* | 3 |
|  |  |  |  |
| **Lacertilia (lizards)** | | | **35** |
| *Anolis* | 4 | *Anolis carolinensis* | 4 |
| *Plestiodon* | 1 | *Plestiodon fasciatus* | 1 |
| *Sceloporus* | 16 | *Sceloporus clarkii* | 3 |
|  |  | *Sceloporus consobrinus* | 2 |
|  |  | *Sceloporus jarrovii* | 5 |
|  |  | *Sceloporus undulatus* | 2 |
|  |  | *Sceloporus virgatus* | 4 |
| *Scincella* | 9 | *Scincella lateralis* | 9 |
| *Urosaurus* | 5 | *Urosaurus ornatus* | 5 |

**Table S1.** Description and count of herptile species processed.

**Table S2.** Pre-processing settings for mass feature detection using MZmine2.

| **MzMine 2.5.3 parameters** |  |  |
| --- | --- | --- |
| Mass detection | MS1 | 1.00E+03 |
|  | MS2 | 1.00E+01 |
| Chromatogram building (ADAP) | min group size in # scans | 3 |
|  | group intensity threshold | 3.00E+03 |
|  | Min highest intensity | 3.00E+03 |
|  | m/z tolerance | 0.001 or 5 ppm |
| Deconvolution (Local minimum search algorithm) | Chromatographic Threshold | 10.00% |
|  | Search minimum in RT range (min) | 0.1 |
|  | Minimum relative height | 10.00% |
|  | Minimum absolute height | 5.50E+04 |
|  | Min ratio of peak top/edge | 2 |
|  | Peak Duration range (min) | 0.01-0.5 |
|  | m/z center calculation | median |
|  | m/z range for MS2 scan pairing | 0.002 Da |
|  | RT range for MS2 scan pairing | 0.05 min |
| Isotopic peak grouper | m/z tolerance | 0.001 or 5 ppm |
|  | RT tolerance | 0.05 min |
|  | Max charge | 2 |
|  | Most representative | lowest m/z |
| Alignment (Join Aligner) | m/z tolerance | 0.001 or 5 ppm |
|  | Weight for m/z | 75 |
|  | Weight for RT | 25 |
|  | RT tolerance | 0.1 min |
| Gap filling (Multithreaded) | Intensity tolerance | 10% |
|  | m/z tolerance | 5 ppm |
|  | RT tolerance | 0.1 min |
